# Quantitative Health Impact Assessment of Environmental Exposures Linked to Urban Transport and Land Use in Europe: State of Research and Research Agenda

**DOI:** 10.1007/s40572-025-00505-7

**Published:** 2025-10-21

**Authors:** James Woodcock, Lambed Tatah, Paulo Anciaes, Zorana Andersen, Ronita Bardhan, Xuan Chen, Audrey de Nazelle, Ulrike Gehring, Stefan Gössling, Marco Helbich, Gerard Hoek, SM Labib, Sasha Khomenko, Haneen Khreis, Danielle MacCarthy, Jennifer S. Mindell, Ismaïl Saadi, Nadja Schweiggart, Cathryn Tonne, Meelan Thondoo, Honorine van den Broek d’Obrenan, Belen Zapata-Diomedi, Mark Nieuwenhuijsen

**Affiliations:** 1 MRC Epidemiology Unit, University of Cambridge, Cambridge, United Kingdom; 2 Bartlett School of Environment, Energy, and Resources, University College London, London, United Kingdom; 3 Section of Environmental Health, Department of Public Health, University of Copenhagen, Copenhagen, Denmark; 4 Sustainable Design Group, Department of Architecture, University of Cambridge, Cambridge, United Kingdom; 5 Institute for Risk Assessment Sciences, Utrecht University, Utrecht, the Netherlands; 6 Centre for Environmental Policy, Imperial College London, London, United Kingdom; 7 School of Business and Economics, Linnaeus University, Kalmar, Sweden; 8 Department of Human Geography and Spatial Planning, Utrecht University, Utrecht, the Netherlands; 9 Barcelona Institute for Global Health (ISGlobal), Barcelona, Spain; 10 Queen’s University Belfast, Belfast, United Kingdom; 11 Research Department of Epidemiology & Public Health, University College London, London, United Kingdom; 12 Department of Socioeconomics, University of Hamburg, Hamburg, Germany; 13 C40 Cities Climate Leadership Group, London, United Kingdom

## Abstract

In this article, we summarise recent developments, identify gaps, and propose a research agenda for quantitative health impact assessment (HIA) of environmental exposures linked to urban transport and land use. This is based on a workshop of 30 experts, complemented by targeted literature identified by participants to illustrate the state of research and practice gaps. The practice of quantitative HIA in urban transport and land use interventions covers a diverse range of methods, models, and frameworks. The selection of an appropriate model depends upon the use case, i.e., the research question, resources and expertise, and application. The plurality of models can be a strength if differences are explicit and their implications are understood. A major gap in most assessments and frameworks is the lack of equity consideration. This should be integrated into all stages of the HIA, considering exposures, susceptibility, disease burden, capacity to benefit, household budgets, responsibility for harm, and participation in the process. Scenarios of environmental exposures in urban transport and land use interventions are often overly simple, while the scenario design process of spatial planning is often opaque. Researchers should specify the involvement of stakeholders and the data, evidence, or behavioural model used to construct the scenario. Recent developments in exposure assessment (remote sensing and modelling) have increased the capacity to conduct HIAs for small geographies at scale. At the same time, advances in simulation have enabled the representation of behaviours at high spatial and temporal resolution. The combination can enable person-centric measures accounting for location, activities, and behaviours, with HIA proceeding ahead of epidemiology. Most HIAs still use Comparative Risk Assessment. This is suitable for estimating the disease burdens of environmental exposures, but more advanced longitudinal methods are better suited for studying interventions. Beyond health outcomes, well-being must be incorporated. The monetisation of health outcomes through welfare economics remains contentious. Representation of uncertainty is increasingly acknowledged. Value of Information methods can inform where new data collection would most efficiently reduce final result uncertainty. In the context of the climate crisis and related environmental limits, methods are needed that consider adaptation alongside mitigation and prevention and test robustness to an increasingly unstable future.

## Introduction

Contemporary urban and transport planning and practices contribute to harmful exposure to air pollution and noise, urban heat islands, and a lack of green space and opportunities for physical activity. It is imperative to understand both the magnitude and distribution of the disease burdens from these risk factors and the potential impacts of urban and transport policies and scenarios to effectively inform advocacy and policy to reduce these burdens. This is increasingly important with the required rapid transition to Net Zero and the failure of the transport sector to decarbonise [1].

The term health impact assessment is used in various ways. This article focuses on quantitative HIA (qHIA) or health impact modelling of disease burden and the effects of scenarios. The term HIA is also defined by the World Health Organization (WHO) to describe a structured approach to studying the potential health effects of a policy, programme, or project on a population, particularly on vulnerable or disadvantaged groups [2]. An HIA following this structured approach might contain a quantitative health impact model. Equally, quantitative HIA might involve all or some of the other stages, but is often conducted as a desk exercise by researchers. In this paper, we refer to HIA as quantitative HIA.

Quantitative health impact assessment (HIA) covers a range of methods to enable a quantitative understanding of the burden of and impacts of changes to the factors that influence population health, such as environmental risk factors and spatial planning [3]. It includes both disease burden assessment of current exposures and the analysis of the potentially preventable burden of future exposures. By comparing the outcomes of alternative exposures to environmental stressors (e.g. air pollution, noise), behaviours (e.g. physical activity) and potential policy scenarios, these assessments are often intended to support scientific and public understanding and inform policy making.

This paper is primarily intended for researchers working in quantitative HIA and related fields, including those developing methods in exposure assessment, epidemiology, and modelling. At the same time, by highlighting conceptual frameworks, scenario design, and communication challenges, we also provide insights relevant to practitioners and policymakers engaged in urban and transport planning. We therefore adopt a broad perspective: synthesising recent research developments for a scientific audience, while also indicating where these advances can support decision-making in practice.

The last 15 years have seen an increasing number of studies estimating the disease burden of transport and urban systems [4, 5]and modelling the impacts of scenarios [6–8]. Increasingly, there are more studies in the Global South [9, 10].

While these studies vary in conceptual framework, scenario design, choice of health pathways from transport and urban planning, exposure representation, choice of outcomes, and HIA methods, there are many similarities. Perhaps, most notably, there has been inadequate consideration of equity [6].

We organised a workshop to identify the existing knowledge base, outline research gaps, and propose a comprehensive research agenda for HIA in Europe in the next 10–15 years. The specific objectives of the workshop were to:

- Identify HIA frameworks, models, and scenarios for urban and transport planning.
- Seek input on strategic research areas, methods, and gaps in terms of metrics, exposure assessment, health pathways, and health, social and economic impact calculations.
- Propose a research agenda for urban and transport HIA in Europe in the next 10 to 15 years.

The next section, Methods, describes the approach. The following section on Frameworks, summarises conceptual frameworks that link urban and transport planning with health, highlighting their scope and limitations and the importance of equity. The fourth section, Urban and Transport Planning Scenarios, discusses the definition of scenarios, considering climate change, time, and stakeholders’ participation. Section five, Environmental determinants and disease burden pathways, identifies the practices and gaps in the most important pathways: air pollution, noise, physical activity, blue and green space, and heat. Section six, Personal Exposure and Behaviour, highlights the increasing potential for assessment of personal exposures (both measured and modelled) and behaviour. Section seven, Health Impact Modelling Methods and Wider Metrics, presents the most commonly used methods of Comparative Risk Assessment (CRA), life tables, microsimulation methods, well-being and economic outputs, and methods for handling uncertainty and equity in HIA. The final sections, discuss reproducibility, summarise and outline a future research agenda.

## Methods

The Horizon Europe-funded project, “Urban Burden of Disease Estimation for Policy Making (UBDPolicy)”, aims to improve the estimation of health and socio-economic impacts of urban and transport-related environmental stressors, advance methodological approaches and foster their acceptance as good practices, thereby strengthening evidence-based policymaking [11].

In July 2023, we organised a three-day workshop in Cambridge, UK, with 30 participants specialised in various aspects of HIA of urban and transport planning in European cities. The participants included senior and early career researchers and practitioners. Participants were selected based on expertise in exposure assessment (air pollution, noise, physical activity, heat, green/blue space), health impact methods, cost-benefit analysis, well-being, and advanced modelling. Most were members of UBDPolicy; selected external experts were invited for complementarity. Author affiliations provide further detail.

The invited participants had expertise in key components of developing a quantitative HIA: health impact methods and exposure assessment (air pollution, noise, physical activity, heat, green and blue spaces). Invited participants also included experts in cost-benefit analysis, well-being, systems thinking, and advanced modelling methods such as agent- and activity-based models. The workshop consisted of presentations, plenary sessions, and small group discussions. The workshop outputs were then augmented and refined to define the state of research, research gaps, and agenda by a smaller working group over 2023 and 2024. This paper synthesises outputs from the workshop, complemented by targeted literature identified by participants to illustrate the state of research and practice gaps. It is not a systematic review but an expert-driven perspective.

## Conceptual Frameworks

The outcomes of HIAs depend on how issues are framed and prioritized, e.g., identifying the critical pathways from transportation systems to health outcomes and selecting the most relevant health metrics. Conceptual frameworks serve as essential tools in this process, enabling the construction of models that map the sequence of events from urban and transport planning scenarios to their direct and indirect health impacts. These frameworks help stakeholders visualize and understand the potential health benefits or risks associated with various planning decisions and how these impact diverse population groups, potentially supporting more informed and health-conscious urban development [12]. Below these frameworks are the quantitative models (e.g., the Integrated Transport and Health Impact Modelling Tool (ITHIM) [13]) used for HIA, which apply a variety of HIA methods (e.g., microsimulation). In some cases, the models are used to produce tools enabling wider use (e.g., the WHO HEAT tool [14]).

We identified frameworks for the relationship between urban and transport planning and health. These frameworks include many of the same risk factors and determinants of health, including individual and societal characteristics, environmental factors, transport and land use policies and practices. We understand risk factors as those that directly affect health, such as exposure to air pollution, and determinants of health as those that influence these factors, such as transport systems affecting air pollution exposure. The frameworks vary in the pathways they emphasise and consider in detail, their scope (globalisation, urbanisation, demographic, economic, or technological changes), and their target audiences (policymakers, researchers and/or urban/transport planners) [15, 16].

Hoskings et al. [17] aimed to develop a generalisable framework that foregrounded health equity building on 94 previous frameworks. They found that climate change and otherdeterminants were included in less than a quarter of frameworks and health equity in fewer than one in ten. They noted that equity considerations require a spatial perspective and integration of financial costs.

Giles-Corti et al. [18] summarised pathways through which urban and transport planning decisions affect health, starting with upstream urban system policies covering transport, land use and urban design, social and health services, education, employment and economic development and housing. The authors linked regional and local planning and design interventions to travel mode choice and daily living outcomes (e.g. access to employment, education, food, health services, social networks) as well as the role of attitudes, preferences, social norms, and mobility needs on transport demands. These, in turn, affect exposure to air and noise pollution, biodiversity loss, exposure to heat, physical inactivity, and other exposure and lifestyle factors, affecting injury and mental and physical disease outcomes. This framework further expands to include transport impacts on emissions, climate change, and extreme climate events like flooding. Notably, the effects of biodiversity loss and the downstream impacts on vector and water ecology and infectious diseases are included. The framework is wide and overlaps with others [15, 19, 20].

The framework developed by Glazener et al. [12] is one of the most comprehensive representations of health pathways. They framed transport as the interaction between land use and the built environment, infrastructure, mode choice, and emerging technologies and disruptors. They identified 14 pathways to morbidity and mortality outcomes, including pathways beneficial to health like physical activity, access and mobility independence, and pathways detrimental to health like air pollution, motor vehicle crashes and heat. They consider effect modifiers at the individual level, including sex, age, and ethnicity, with equity as a cross-cutting theme.

Overall, these frameworks aid in structuring thinking around spatial planning, transportation, and health outcomes and identifying gaps in knowledge and research. However, there have not been evaluations of the effectiveness of the frameworks in changing thinking or practice.

Moreover, quantitative HIA methods and models can only partially represent the complexity inherent in these frameworks. The practical development of quantitative HIA models, particularly of tools, requires specifying the use case(s). The use case should be considered the combination of the problem (research or policy question) and the resources that can be made available to model it.

## Urban and Transport Planning Scenarios

### Scenario Definition

Quantitative HIAs rely on counterfactual scenarios (alternative future scenarios) and compare current conditions (or business-as-usual projections) with these scenarios. Defining these scenarios is a critical step in the HIA process. It shapes the scope of analysis and determines, to a certain extent, the most important health impacts the assessment will evaluate as an outcome of policy options.

In the simplest case, when assessing the burden of a single harmful risk factor, the scenario typically references some minimum level of exposure. This could be the lowest observed exposure, theoretical, or feasible minimum in a given population. Reference levels can be updated based on new empirical exposure estimates, new epidemiology on the harms at lower levels, or international guidelines on what is feasible. For example, the WHO’s recommended guideline value for particulate matter PM_2.5_ recently changed from 10 to 5 micrograms per m [3] annual average, resulting in a significant increase in the reported disease burden of air pollution when the new value was used as the reference case (or counterfactual) [21]. Many HIA studies have assessed the avoided burden of achieving different minimum levels of exposure to air pollution [22, 23].

The situation for determinants of health is more complex, as these typically operate through multiple pathways affecting health. Even for a relatively simple case, such as investing in cycling, there are direct and indirect benefits and potential harms. When considering the totality of transportation and land use harms and benefits, it is unclear how to define a theoretical minimum. The observed minimum is not knowable without a detailed HIA. Defining a feasible minimum requires specifying what a healthy and functionally sufficient transport system and land use would look like [24].

HIAs that have studied transport and urban planning scenarios tend to focus on travel mode shift scenarios. While some studies model the long-term impacts of an intervention (e.g. cycle hire study) [25] or use a behavioural model to estimate potential mode shift [26], effect estimates often come from transport plans in policy documents, which may lack adequate supporting evidence. We recommend distinguishing between modelling the best estimate of the effect of a policy and modelling the impacts of a broader vision. The effect of a policy should be based on evidence or a behavioural model. In contrast, a vision does not require evidence of how it would be achieved, but incorporating spatial and demographic realism [27] can help identify potential trade-offs and inform policy design.

### Transdisciplinary Approaches and Stakeholder Engagement

The scenario development process and the target audience invited to engage in HIAs are often not clearly described [6]. While the best approaches will depend on the nature of the assessment, there are increasing calls for more stakeholder engagement and the critical role of transdisciplinary collaboration in processes of co-design [28, 29]. Ideally, both co-design (participatory HIA) and transdisciplinary approaches are used for scenario building and engagement in HIAs. They enable active participation from stakeholders, including local communities and policymakers, to co-design goals, frame problems, build contextually relevant solutions, and monitor and evaluate impact (C40 Cities Climate Leadership Group, C40 Knowledge Hub, 2019). This allows alliances to be formed across sectors and can generate buy-in so the evidence produced is more policy-relevant and the policy leadership feels more compelled to take action in response to stakeholder needs [28]. This participatory process also facilitates knowledge translation, ensuring that findings are communicated in accessible formats and directly inform decision-making.

Yet, stakeholder engagement in HIAs presents challenges, particularly when utilising participatory approaches. While local communities offer invaluable knowledge and insights, their involvement can sometimes complicate policy implementation. Balancing these contributions with policy goals is a complex process. Evaluation of the gaps and needs of the community and identifying the key impacts to model and prioritise in the policy design can help ensure that the HIA assesses the distribution of impacts and potential equity issues. One of the key challenges is the time and effort required to build meaningful partnerships, which often clash with the tight timelines of policy-making processes. Another challenge can be scalability: an engagement can enrich the development of a small-scale policy, but may miss new challenges and learnings when scaling up the policy.

In practice, HIAs tend to rely heavily on expert input, particularly when rapid decision-making is needed, limiting the extent of broad community participation. Additionally, participatory processes occur within broader contexts of unequal power, resources, skills, and knowledge shaped by ideological and political narratives. Issues of class, race, gender, and geography can influence who participates and how their voices are heard, making the process of engagement highly contingent on these factors. Thus, starting any assessment by mapping communities that may be underrepresented is valuable [30].

The effective communication of HIA findings, particularly from complex modelling to non-technical stakeholders, is crucial. Simplifying complex data into data stories can bridge gaps across departments, ensure insights are shared with a wider audience, and be more compelling to policymakers [18, 31]. While the detailed methods used will often make it hard for non-expert groups to follow the factors considered in the approach, the results, including trade-offs, can be more widely understood. There is often tension between stakeholders wanting simple methods and wanting answers to specific and detailed questions about the impacts on different populations, including multiple influences.

Aligning the timing of HIA dissemination with political cycles, such as mayoral elections, can increase the likelihood of policy influence, as research results may be better positioned to capture decision-makers’ attention. Policymakers often perceive participatory activities as being too resource-intensive [32]. However, well-designed participatory HIAs can save time by pre-empting conflicts and improving stakeholder buy-in from the outset [33].

Technological tools, such as online platforms for data collection and community engagement, offer opportunities to enhance engagement. These tools can make gathering diverse inputs easier and engage stakeholders who cannot attend in-person meetings. However, technology is not a panacea for addressing more profound socio-economic and political challenges. HIA researchers and practitioners should continue to align their work with broader struggles for equity and justice, ensuring that participatory approaches contribute to long-term systemic change rather than merely offering short-term solutions [34].

### Time and System Boundaries

A key decision in designing future scenarios is deciding what to change and hold constant. Many factors will change, and the future is inherently uncertain [35]. Change can be exogenous (e.g. changes in obesity assumed based on background trends) or endogenous (e.g. changes in obesity modelled as dependent on changes to the food and physical activity environment).

There is often a trade-off between detailed realism and analytic clarity for exogenous changes. For instance, in a mode shift scenario, one could consider how increasing electric vehicle take-up or improving health status might reduce the benefits. However, including these elements adds uncertainty and can make interpreting results more challenging. Thus, many scenarios are considered alternative versions of the present, studying marginal change.

Even when projections are made, these usually cover only a few years. Long-term projections are difficult, and short-term thinking is encouraged by economic theory and practice (with welfare economic-based discounting of the future) and political timeframes. However, in the context of the Anthropocene, there is a strong argument for the development of impact assessment methods that are appropriate for much longer periods.

With climate change upon us, it is important to factor adaptation, resilience, and mitigation under increasingly unstable futures in the design of scenarios. Warming relative to the preindustrial period is projected to be between 1.6 °C and 2.4 °C (under IPCC scenarios SSP1-1.9 to SSP5-8.5) between 2041 and 2060, depending on global mitigation efforts and climate sensitivity [36]. Heatwaves, floods, wildfires, and other extreme weather events will impact urban mobility. The number and/or size of areas where people can be active outdoors may decrease substantially. Adaptation efforts need to consider the contribution of transport infrastructure to urban heat islands, the resilience of the electrical system to demand during extreme heat and the impacts of sea level rise on active and public transport infrastructure [37]. Globally, major migrations will require highly adaptable urban planning and social systems and overcoming ethnonationalist ideologies. Scenarios should test robustness under varying future climate conditions, considering not just the average but the risk of catastrophic events, recognising that many impacts will play out over hundreds of years.

## Environmental Determinants and Disease Burden Pathways

This section considers air pollution, noise, green/blue space, heat, and physical activity as pathways via which transport and urban planning impact health. After defining each pathway, we consider challenges in measurement and/or modelling sources, disease burdens, and issues in their application to HIA. These pathways were selected for inclusion due to existing evidence from systematic reviews and meta-analyses on their causal mechanisms related to health outcomes and the role of urban and transport planning in exposure to these pathways. Future workshops in UBDPolicy will recommend the choice of exposure-response functions for HIA. Additional pathways discussed at the workshop were community severance and traffic injuries and falls.

### Air Pollution

#### Burden and Pathways

Air pollution is the largest environmental disease burden and ranks highly among risk factors for disease [38]. The European Environment Agency (EEA) states that PM_2.5_ caused 239,000 premature deaths in Europe in 2022 (using zero as reference/counterfactual). Similarly, NO_2_ and O_3_ were linked to 48,000 and 70,000 premature deaths in 2022, respectively, according to the EEA 2023 estimates [39].

There is no safe level of air pollution exposure, and the major message of the new WHO guidelines is that air pollution is harmful at even lower levels than previously realised. This, alongside new meta-analyses [21, 40–43] showing stronger exposure-response functions for PM_2.5_ (steeper slopes of concentration-response functions and increased evidence strength), has led to substantial increases in estimates of the burden of air pollution.

Air pollution emissions come from many anthropogenic and natural sources, and there are different approaches to estimating the contribution of different sources to concentrations. For Europe, the EEA has provided estimates of source contributions. Many sources contribute to PM_2.5_ and PM10, while road transport is the leading source of nitrogen oxide (NO_x_) emissions [44]. The contribution of a source to emissions does not necessarily agree with the contribution to concentrations. This discrepancy arises because dispersion, chemistry, and geography influence concentrations differently across pollutants and sources.

There is strong evidence from epidemiological studies, supported by toxicological evidence, linking air pollutants to many health outcomes, including e.g. cardiovascular and respiratory disease, lung cancer, low birthweight, diabetes, dementia and related premature mortality [45, 46]. The greatest burden is from PM_2.5,_ but there is also strong evidence of harm from nitrogen dioxide (NO_2_), black carbon and ozone (O_3_). Health effects are related to short-term (days, hours) and long-term exposure (years or multiple years). While there is potentially differential toxicity for distinct PM sources and composition, the evidence is not conclusive [47]. Therefore, PM_2.5_ remains a useful exposure metric for HIA of generic air pollution [47].

#### Measurement and Modelling

##### Modelling Concentrations and Exposure

Assessment of long-term exposure to ambient air pollution for epidemiological studies remains challenging [48], but it is necessary to derive exposure/concentration-response functions. Early cohort studies characterized exposure by assigning the average concentration measured at a few urban central sites to each person within the city [49]. However, within-city spatial contrasts may be even larger than the between-city contrast, particularly for combustion-related pollutants, such as NO_2_, black carbon and ultrafine particles, depending on the location of sources and patterns of dispersion [50]. 

To characterize intra-urban contrasts, approaches beyond direct monitoring have been developed, including exposure indicator variables (e.g. traffic intensity at the residential address or distance to a major road), interpolation methods (e.g. kriging, inverse distance weighting), dispersion models and land-use regression models [51]. Also, due to insufficient surface monitoring, satellite data is increasingly being used to estimate air pollution exposure. Surface monitoring data are typically spatially sparse, whereas models and satellite data are spatially more complete at the expense of more uncertainty. 

Land use regression (LUR) models are empirical (regression) models combining monitoring of air pollution at a limited number of locations and collection of variables via geographic information systems (GIS), which can potentially predict the measured spatial variation [51, 52]. Mobile and short-term monitoring campaigns have been conducted to provide the high temporal and spatial variability required to model combustion-related particles [53, 54]. LUR models may be the method of choice if there is significant uncertainty about emission factors or physical-chemical transformation processes. Still, their empirical nature makes them less transferable to other areas and is not useful in predicting policy impacts or scenario changes [55]. 

Dispersion/chemical transport models (DCTMs) are deterministic models, using physical and chemical knowledge to model the dispersion and chemical transformation of emitted pollutants from sources. DCTMs have frequently been applied in epidemiological studies, especially in European studies [56–59]. Some studies have gone down to individual addresses [56], while other models for PM_2.5_ were at a larger spatial scale of 1 km^2^ or above [57, 59]. More effort and expertise are needed to collect input data compared with land use regression models. The quality of the input data is a key determinant of the performance of a DCTM. 

A wide variety of models exists that differ in the spatial scale (e.g. street, urban, regional, continental, or global) and the processes they include (only dispersion versus dispersion plus chemical transport). Recognizing the limitations of any single method, hybrid models incorporate multiple methods in one framework. 

Air pollution surfaces have been increasingly available on a large scale over many years. For example, Shen et al. developed hybrid air pollution models predicting annual and monthly air pollution exposure surfaces at a higher spatial resolution (25 × 25 m) from 2000 to 2019 for PM_2.5_, PM_10_, NO_2_ and O_3_ across Europe [60]. They used geographically weighted regression to explain (temporally) variations in air pollution concentrations measured at AIRBASE monitoring sites using several predictor variables, including satellite data, chemical transport model estimates, road network and land use data. 

However, these surfaces still do not account for exposure in transport microenvironments, which can vary considerably. A recent review and meta-analyses [61] found large variations across studies and pollutants globally. The tendencies for users of motorized modes were to be more exposed to NO_2_ and less exposed to particles compared to pedestrians, and generally, similar exposures compared to cyclists for particles (but insufficient data for NO_2_). Bus riders had larger exposure than pedestrians and cyclists for most pollutants except ultra-fine particles. An earlier review of European studies showed a clearer pattern of pedestrians being the least exposed to various pollutants, cyclists and bus riders, and car users being the most expose [62]. 

##### **Source Apportionment**

For scenarios based on achieving some minimum level of air pollution, estimates of the concentrations are sufficient. However, to estimate how a policy might change concentrations, it is also necessary to have models sensitive to changes in emissions from different sources of air pollution concentrations. Emission reduction impacts are the most widely used, based on chemical transport model simulations. They estimate source contributions to air pollution based on concentration differences from modelling all emissions versus reduced emissions for specific regions or sectors [63]. Given that running a full Chemical Transport Model simulation is computationally intensive, several simplified approaches that reduce computational time have been developed, such as the FASST [64–67], at the national, regional and/or urban scales.

#### HIA

There are many HIAs on air pollution on the global, European, and city scale [68–70]. These are normally based on residential outdoor exposure and have benefited from the transformation in the ability to estimate concentrations at a high spatial resolution. However, this is typically converted to an annual average, ignoring spatial and temporal variability. This ignores exposure away from the house, including elevated exposures while travelling due to proximity to traffic and heightened ventilation rates when walking or cycling.

While evidence suggests that the underestimation of relationships is relatively small for epidemiological analyses [71], these differences could be more important for HIA, particularly when considering equity. Secondly, most of the time at home is spent inside, not at the front door. A recent modelling study integrated outdoor and indoor concentrations in different microenvironments, accounting for variations in time-activity patterns, and found children’s exposure to be primarily driven by indoor sources in the home [72]. Another issue of increasing relevance is the harm of different constituents of PM; particularly with the transition to electric vehicles, we need to better understand the harms related to brake, tyre wear, and resuspension.

Another methodological challenge is the treatment of multiple pollutants. Most HIAs have considered single pollutants in isolation, yet people are exposed to complex mixtures. Multi-pollutant models can, in principle, better reflect combined exposures; however, they require careful attention to collinearity between pollutants and to the interpretation of effect estimates. As the evidence base grows, particularly for emerging constituents such as brake and tyre wear particles, multi-pollutant approaches will become increasingly crucial for advancing HIA.

### Noise

#### Burdens and Pathways

At its broadest, noise encompasses all undesired and harmful sounds. However, much of the evidence on harms has focused specifically on transport noise, often on specific modes [73].

In urban environments, the main source of environmental noise is transport. Road traffic noise is the predominant source, followed by railway, aircraft, and industry noise. In Europe, the number of people exposed to long-term noise levels of 55 dB or higher is estimated to be 113 million for road traffic noise, 22 million for railway noise, 4 million for aircraft noise and fewer than 1 million for industrial noise [73].

Studies have linked environmental noise with health effects, including sleep disturbance, annoyance, cardiovascular and metabolic disease, adverse birth outcomes, cognitive impairment and poor mental health and wellbeing [74–80], with strong evidence linking noise to ischaemic heart disease [78] (Kempen et al., 2018). A recent umbrella review also indicates strong associations with all-cause mortality, all cardiovascular diseases and diabetes. The evidence is stronger for road traffic noise compared with other sources, such as railway and aircraft noise, which might be related to the availability and quality of studies on the topic [81].

The risk for adverse health effects due to environmental noise tends to increase from 45 dB L_den_ or below for high annoyance, cardiometabolic outcomes and mortality, and from 40 dB L_night_ for high sleep disturbance [81].

In Europe, an estimated 22 million people suffer from chronic high noise annoyance, and 6.5 million people experience high sleep disturbance due to environmental noise, leading to 12,000 premature deaths and 48,000 new cases of ischemic heart disease each year [73]. Noise is considered the second major environmental cause of adverse health outcomes in Western Europe after PM [82].

#### Measurement and Modelling

In Europe, the member states deliver environmental noise data under the Environmental Noise Directive (END, Directive 2002/49/EC) every 5 years. The member states produce strategic noise maps and calculate the number of people exposed to each noise source [73]. In addition, strategic noise maps are produced and published in Europe through the Environmental Noise Directive or local city councils. However, the models used to model noise exposures, the coverage, and the data quality are hugely variable. It is necessary to carefully evaluate the underlying noise modelling approach, data format and noise exposure assessment (e.g. categorization or street-level/building façade exposure) to ensure that data are suitable for HIA [83]. In EXPANSE, road traffic noise was modelled across Europe using the CNOSSOS_EU method [84, 85], representing traffic on all roads. Some studies have also started using mobile measurements of noise [86].

Individuals’ exposure to noise will depend on the proximity to the noise source, building height, residential building features, including bedroom orientation, window types and shielding materials, and behaviour such as closing windows or wearing earplugs [83]. Barriers can also reduce exposure from high traffic volumes, though these reflect noise and obstruct views. New materials (such as sonic crystals) and nature-based solutions are being tested [87]. Thus, incorporating information such as population distribution in residential buildings and building floors and exposure at the building façade can refine exposure assessment and help reduce exposure.

#### HIA

Environmental noise HIAs are less common than air pollution [83]. Noise assessment is typically at the highest exposed building façade. While it might account for the shielding of buildings, it does not account for how noise is experienced in the home or for exposures while travelling, outside, or inside other buildings.

### Heat

#### Burden and Pathways

While this section focuses on heat, cold exposure also remains a relevant disease burden in Europe, particularly among disadvantaged groups with poor housing and energy insecurity.

Globally, the past decade has recorded the warmest years. The year 2022 recorded the highest number of extreme heat hours, and 2024 was the hottest year on record, where global temperatures exceeded 1.5 °C above pre-industrial levels for the first time in history. Over the past 30 years, the frequency of exposure to extreme heat has grown, adding up to an extra 26 days of extreme heat last year [88].As global temperature increases above 1.5 °C, short-lived but highly intense heat events distinct from seasonal averages will become more intense and frequent.

Extreme heat becomes lethal when high temperatures are combined with high humidity such that the body cannot release excess heat through the evaporation of sweat. At the same time, long-term heat exposure can lead to chronic diseases [89]. Meta-analyses show that all-cause cardiovascular and respiratory illnesses are the main causes of death during heatwaves [90, 91]. Epidemiological studies have shown that exposure to high ambient temperatures is associated with premature mortality, cardiovascular and respiratory morbidity, children’s mortality, and hospital admissions [92–96]. Temperature and mortality are associated during extreme events, such as heat waves, and at less extreme temperatures [97, 98]. Recent studies in Europe during the summer on heat-related deaths indicate 114 (95% CI = 69–160) heat-related deaths per million population, with a 56% higher rate of heat-related deaths in women than men [99].

Human vulnerability and adaptive capacity play a significant role. People have different heat tolerances based on acclimatisation, thermal history, social cooling practices and gender. However, these factors are not captured using current epidemiological studies [100, 101]. Inequality of heat-adaptative infrastructure, like shade, increases hazards. While evidence on heat hazard sources is established, data on heat exposure, vulnerability, adaptive measures, and human heat resilience are still emerging.

Variation in urbanization patterns leads to differential microclimates, driven by differences in urban morphology, green infrastructure such as trees and vegetation, and blue spaces like water bodies. The trapping of solar radiation due to these variations in urbanisation patterns results in localized heat stress, which is further exacerbated by anthropogenic heat sources, including vehicular emissions and building exhaust. These interlinked factors frequently modulate and intensify exposure to extreme heat [102]. Recent studies have demonstrated that the cooling efficacy of cities depends on urban morphology, background climate zones, and tree traits. In Tropical countries, high-density urban forms and evergreen trees can cause nighttime heat stress [103]. In urban areas, the trapped heat during the day that fails to escape into the atmosphere causes urban heat island (UHI) effects. These UHI effects are further exaggerated by various high thermal mass materials used in the construction of buildings and infrastructure. Thereby intensifying heat stress in certain areas of the city.

#### Measurement and Modelling

Heat exposures are mostly estimated at low spatial resolutions, while the heat-health impacts are individual. However, temperature measurement is improving. Bussalleu et al. [104] applied a 2-stage model to predict daily mean, minimum and maximum temperature at a 1 × 1 km spatial resolution across Europe from 2003 to 2020. The first stage produced daily gap-filled Land Surface Temperature from Moderate Resolution Imaging Spectroradiometer instruments aboard the Aqua and Terra satellites. In the second stage, this was combined with European Centre for Medium-Range Weather Forecasts (ECMWF) meteorological variables, land use and elevation data to explain spatiotemporal variance in measurement data across more than 5000 European weather stations.

#### HIAs

European HIAs are assessing the impacts of reduced exposure to heat on mortality. These studies used spatial maps of heat exposure based on daily mean temperatures to estimate heat-related impacts in European cities [98]. Future work needs to consider microclimates, adaptation, vulnerability, and resilience.

### Green and Blue Spaces

#### Burden Pathways

There is no universal definition of green or blue spaces [105], with different definitions depending on the subject area and use case. Green and blue spaces generally refer to areas of vegetation and water within urban environments, often designed for recreational, aesthetic, and ecological purposes. Green spaces mostly include parks, gardens, and natural reserves, while blue spaces encompass ponds, lakes, rivers, and fountains [105]. Much of the evidence refers to green spaces or unspecified ‘Nature exposure’, making disentangling the role of green versus blue space difficult.

Epidemiological studies indicated that green and blue space exposure is associated with positive health conditions through multiple pathways [106–108]. Some are direct from observing greenspace, some require agency (e.g. physical activity and community activities), while others are effect modifiers on exposures to other pathways (e.g. air pollution, urban heat island). Greenspace is important for physical and mental health (e.g., stress recovery) [109, 110]. However, these interacting pathways make isolating independent epidemiological relationships for integrated assessment in HIA more difficult. Much of the evidence is cross-sectional, with meta-analyses combining cross-sectional and longitudinal studies [107, 111], except for Rojas-Ruedas et al. [112], including cohort studies only. The evidence is strongest for all-cause mortality, with less good evidence for morbidity outcomes.

Based on the WHO standard for greenspace access in residential areas, Barboza et al. [113] estimated that in 1027 European cities, higher residential greenspace exposure could prevent 42 968 (95% CI 32 296–64 177) premature deaths annually per 100,000 inhabitants-year.

#### Measurement and Modelling

Greenspace exposure-response functions are mostly based on greenspace availability exposure (e.g., satellite-derived vegetation indices such as Normalised-Difference Vegetation Index (NDVI), percentage greenspace) around the home [112, 114, 115]. NDVI measured by diverse satellite images (e.g., Landsat) has been traditionally used as an indicator of greenspace across Europe. In addition, many studies have measured greenspace exposure regarding accessibility to publicly open areas and eye-level visibility of greenness around the home environment using street view imagery [114, 116]. High-resolution input data (e.g., satellite images) may be translated into different exposure-response functions.

#### HIA

The most common greenness exposure assessment methods used in HIAs are limited to using a buffer around the home. These ignore exposure beyond the home environment [106], and there is no consensus about what buffer distance should be used for such exposure assessment [114].

It is unclear if short versus long-term exposure is more important for health benefits [117, 118]. For HIA, the reliability of the exposure metrics and exposure-response functions might be influenced by these factors (e.g., buffer distance, exposure contexts, and temporal aspects).

In addition, greenspace exposure often indicates nonlinear relations with health outcomes. Usually, these relations are mediated or moderated by other environmental exposures such as air pollution, noise, and heat [119, 120]. Similarly, socioeconomic position may confound these associations, as greener areas often differ systematically in deprivation levels, housing, and services. HIA for greenspace exposure thus requires critical consideration for multi-exposure interactions and nonlinearity in the effect of greenspace exposure.

### Physical Activity

#### Burden and Pathways

Much physical activity is achieved through activities of daily living, notably walking and cycling. The built environment shapes the likelihood of these behaviours, and these behaviours further affect people’s exposure to air pollution, noise, and green space.

The evidence is strongest for non-occupational physical activity, and there are well-established dose-response relations of cohort studies for self-reported physical activity that can be used in HIA, including reducing the morbidity and mortality risk from disease outcomes such as cardiovascular disease, diabetes, dementia, depression, and several cancers [121]. The evidence consistently indicates a strongly non-linear relationship, with the greatest benefits from increasing activity amongst those least active. Beyond 300 min per week of moderate to vigorous physical activity, benefits on most health endpoints are likely small, apart from weight-related outcomes. The benefits appear to be even greater for objectively measured physical activity [122].

Estimates of the burden of physical inactivity vary depending on the relative risks used and counterfactuals considered. One study estimated the burden at 6.2% of all-cause mortality for Central and Eastern Europe and 9.3% for high-income Western Countries [123]. In contrast, estimates from the Global Burden of Disease study are much smaller [124], with physical inactivity accounting for around 3.3% of all deaths in the European Union (approximately 125,000 deaths, corresponding to nearly 2 million DALYs) (GBD 2019 Risk Factors Collaborators, 2020).

#### Measurement and Modelling

Traditionally, physical activity is measured using self-report surveys to capture habitual behaviour. However, self-report measures correlate poorly with objectively measured physical activity [125], and several options are available for quantifying activity behaviours with wearables. These measurements provide time-stamped information about physical activity behaviours, which, combined with time-stamped geolocation data, allow studying dynamic interactions between environmental exposures and physical activity. As dose-response functions for objectively measured PA increasingly become available, new opportunities for HIA are available [126].

Walking and cycling behaviours can also be captured as trips from travel surveys. However, in these surveys, trips are usually recorded on one day and thus do not capture intraday variability in behaviour, although this can sometimes be inferred from questions on the regular use of modes.

#### HIA

There are many HIAs of physical activity, mostly assessing active transport and urban scenarios, and these use a wide range of dose-response functions [6]. While dose-response functions for walking and cycling exist and are used in many HIAs, e.g. the WHO HEAT tool [14], the burden or impact of interventions more realistically depends on the total physical activity of the relevant population. Studies have incorporated total physical activity using categorical exposures matching categorical relative risks [127]. Incorporating the non-linear dose-response functions in HIA requires estimates of the distribution of baseline activity levels across multiple domains, either from measurement or imputed. While physical activity is directly measured at the individual level for cohort participants, estimation for whole populations requires imputation from surveys using spatial microsimulation (Smith et al., 2021) or potential measurement at scale with the widespread, if biased, uptake of wearables and high uptake of mobile phones [128].

## Personal Exposure and Behaviour

### Personal Exposure

Most HIAs of environmental exposures use aggregated exposure assessments based on people’s place of residence. An aggregate area-based approach only allows the analysis of inequalities based on the average socio-demographic composition of an area. It also ignores variability based on behaviours, that people spend only a fraction of their daily life at home, and that exposure levels are often higher while travelling.

Microsimulation methods based on observed or modelled data allow the representation of variability of behaviours and, hence, exposures for people in a given area. Microsimulation can be aspatial (e.g., for tax and benefit simulations [129]) or spatial.

Generating person-centric exposures based on locations and activities requires linking highly temporally and spatially resolved environmental data with observed or modelled data on people’s locations and activities [130]. While for the large epidemiological studies needed for robust estimation of the exposure-response functions, it has not been possible to measure behaviours at a sufficient scale, a recent review of smaller studies found a good correlation between residential and time-activity-based air pollution exposure (*R* >0.8) leading to around 9% to 30% potential underestimation of effects [71]. However, inequalities in exposures by socioeconomic groups were greater with time-activity-based exposure, and the results did not consider differences in the ventilation rate. Results may also differ for other exposure types.

### Dynamic Exposure Assessment Using GPS-based Methods

While self-reported travel surveys and activity diaries are only partially capable of providing the required information (e.g., recall bias, lack of route-specific data), geo-technologies such as Global Positioning System (GPS) enabled smartphones are a viable means to collect spatiotemporal mobility data in a potentially unobtrusive way [106].

Several challenges must be overcome to realise the potential of dynamic exposure assessments for HIAs. First, besides technical constraints (e.g., signal loss, battery life), respondents are typically reluctant for privacy reasons to participate in GPS-based studies, resulting in small sample sizes that are not representative. This occurs in a context in which personal data, including location tracking and activity participation, has become a major commodity for marketing, with control centred on a few global firms. Secondly, methodological guidelines to harmonise data collection and processing are needed to support study comparability.

Key research questions include how locational tracking best can be integrated with other sensing technologies capturing biomarkers (e.g., electrodermal activity) and physical activity (e.g., accelerometers); how spatiotemporal resolved activity spaces can assess exposure accumulation, duration and sequences using longitudinal tracking; and for which populations do dynamic exposures substantially different from home-based assessments.

### Microsimulation of Personal Exposure

While measuring the whole population is extremely difficult for researchers, simulation is more feasible. While environmental HIAs have tried to improve the spatial representation of exposures and increasingly overlay detailed population data, they still tend to apply to population averages rather than representing individuals. This constrains the HIA’s ability to gauge how different societal sectors are affected by and respond to proposed interventions. However, microsimulation modelling offers the potential to represent population variability better.

The term quasi-microsimulation is sometimes used to describe the microsimulation of exposure assessment combined with an aggregated health impact calculation. Either full or quasi-microsimulation requires a real or synthetic population of individuals. Typically, collating and then using comprehensive data on real populations is not practical, ethical, or legal; hence, synthetic populations are the best alternative. Synthetic populations are created to represent a complete, disaggregated population by combining a sample of disaggregated members to match key distributions for the entire population [131]. These key distributions can be at the household, person, or dwelling level and can be aggregated at different geographical resolutions.

The process of synthesizing a population has two main phases: optimization (fitting) and allocation. The first phase fits a disaggregated sample of agents to aggregated constraints, while the second phase replicates actual agents for the synthetic population using probabilistic selection.

The Iterative Proportional Fitting procedure is a well-established algorithm for fitting [132]. However, it can handle only one level of aggregation and geographical resolution at a time. Iterative Proportional Updating [133] is an evolution of Iterative Proportional Fitting that calculates weights for each microdata record and can handle multiple levels of control attributes simultaneously [134, 135].

### Behavioural Modelling of Exposures

With a synthetic population, environmental attributes can be assigned to diverse individuals. Agent-based models can be particularly useful when these individuals need to interact or learn. Agent-based models are microsimulation models that use autonomous agents to represent individuals or entities within a defined environment. These agents follow specific rules and interact with each other and their surroundings, leading to emergent behaviours and system-wide patterns. Agent-based models are utilized in various fields to study complex systems and predict outcomes based on individual actions and interactions.

Traditionally, transport models aggregated the movement of people, but agent-based models and activity-based models represent individuals. Activity-based models are specific agent-based models developed in transport research to represent individuals’ daily travel behaviour and activity patterns.

Agent-based model network assignment tools like MATSim offer high temporal and spatial resolution. MATSim represents synthetic individuals navigating a study area to accomplish their planned activities, integrating behaviours and environments. By simulating individuals, MATSim captures multiple transport and environment-related pathways, such as air pollution emissions and exposures, noise, and traffic injury risk, down to the specific street segment and second. These outputs can be used to derive exposures for the simulation’s individuals, representing spatiotemporal variability in exposures and behaviours of heterogeneous individuals. These methods could also be used in epidemiological studies to estimate personal exposure better and derive aetiological exposure-response relations [104].

In a Munich case study, air pollution, injury risk, and physical activity were represented for a synthetic population across a long-term land use model, a travel behavioural model, and a network assignment model [26]. While their application to HIA is still in its early stages, transport-derived agent-based models are a highly flexible approach for the inclusion of multiple exposures and detailed representation of heterogeneous populations in realistic environments and hence health determinants (e.g. transport system as a determinant of exposure to air and noise pollution).

## Health Impact Modelling Methods and Wider Metrics

In this section, we consider the main methods used to calculate health impacts (Comparative Risk Assessment, life tables and microsimulation) and then consider well-being and welfare economic analyses.

### Comparative Risk Assessment

Comparative Risk Assessment (CRA) typically uses the population attributable fraction (PAF) to estimate the proportion of burden attributable to exposures. CRA can be used as a general term or specific method. As a general term, it is used to compare the population risk for a specific health outcome when exposure to the agent causing the disease shifts from a baseline scenario to an alternative scenario. As a specific method, it involves multiplying deaths, years of life lost, incidence, or less often years lived with disability and disability-adjusted life years by the potential impact fraction [136], which measures the proportional change in diseases due to changes in risk factors, to produce a change in the metric of interest. Using the term with the second meaning, CRA is a widespread method: it is relatively simple and produces results that appear easy to understand.

While comparative risk assessment is designed primarily to analyse the burdens of risk factors, it is also used to represent the effects of interventions. However, the fact that Comparative Risk Assessment does not include time steps becomes more problematic here. This means it cannot address competing causes of death. For instance, if someone does not die of a disease at a given time, they will die from another cause later in life [137]. Therefore, the Comparative Risk Assessment estimate considers the first-order or direct effects of a change in exposure but not the second-order or indirect effects resulting from a change in mortality.

When presenting results as lives saved or premature deaths prevented, it is hopefully evident to all that death is only postponed, not prevented indefinitely. However, when studying incidence, the implications are more complex. For example, if reducing air pollution lowers the risks of heart attacks and dementia, a reduction in heart attack mortality could result in more older people and potentially more cases of dementia, even if the age-specific risk of developing dementia is lower.

### Life Tables

Life tables have a long-standing history in actuarial science. Unlike Comparative Risk Assessment approaches, they can estimate life expectancy and are widely used to assess the long-term effects of interventions. Basic life tables consider the annual transition probability from being alive to dead.

More advanced methods, such as multi-state life tables, build on life tables by including multiple states (e.g., alive, dead, with disease). These advanced models are more complex to parameterize, and the number of necessary transition probabilities increases exponentially with the number of states.

The proportional multi-state life table method was developed [138, 139] to reduce this complexity. This simplifies assumptions about the independence of states and consists of state transition models to estimate an individual’s or cohort’s probability of developing diseases, dying from them, and dying from other causes at different ages [140]. It allows for the simultaneous modelling of multiple diseases and includes a temporal component by modelling individuals or cohorts over time. Transport and health models have applied it at a cohort level by simulating population groups by age, sex, and ethnicity over time [141, 142].

### Microsimulation of Health Outcomes

Microsimulation has been applied at two stages in health impact modelling: exposure assessment and impact calculation. Suppose microsimulation is applied just at the exposure stage, referred to as quasi-microsimulation (see above). In that case, it can represent how exposures but not diseases (multimorbidity) vary by population subgroups and are clustered (e.g. smoking and alcohol vs. COPD and heart disease). Full microsimulation of exposure and impact calculation can represent variability in susceptibility and heterogeneity, clustering of exposures, and dependence on diseases. Because of the assumptions of independence, the proportional multi-state life table cannot represent the risk of developing diseases and dying from diseases from multimorbidity.

Microsimulation models are highly flexible and can account for differential event rates based on multiple factors. National agencies commonly publish data for all-cause mortality, though usually broken down by a limited number of socio-demographic variables. For disease incidence and case fatality, estimates are often based on indirect data such as prevalence and cause-specific mortality. In these cases, statistical methods can be used to optimise available data and inform a microsimulation model while describing the associated uncertainty [143]. Full microsimulation models are increasingly used for studying risk factors [144], including air pollution [145]. 

### Well-being

The assessment of health impacts related to urban and transport planning has predominantly centred on mortality, morbidity, DALYs, years of life lost, quality of life, and their corresponding economic valuations. However, the WHO has long defined health as the state of physical, mental, and social well-being that enables people to cope with the stresses of life, realise their abilities, learn well and work well, and contribute to their communities (based on the WHO definitions for health and mental health [146, 147]. Nonetheless, HIAs of urban and transport planning typically do not include well-being.

Subjective well-being, happiness, or emotional well-being assesses the pleasure/pain continuum. It comprises three main components: life satisfaction, the presence of a positive mood, and the absence of a negative mood (hedonic well-being). Well-being also emphasizes living according to one’s true self, engaging in activities that align with personal values, and fostering growth and development (eudaemonic well-being) [148]. Well-being economists use metrics such as well-being-adjusted life years to incorporate well-being aspects into economic evaluations [149].

Urban and transport planning can affect the positive aspects of well-being, such as resilience and life satisfaction (e.g., by facilitating social interaction, physical activity, and restorative processes) [150]. Travel for work and other purposes impacts subjective well-being in ways not fully captured by “travel satisfaction” metrics, including access to activities, emotional responses, and physical activity levels. These travel-related factors also affect other areas of life, such as leisure, work, health, and residential well-being.

Several measurements and questionnaires for subjective well-being include life satisfaction, e.g., the WHO-5 well-being index [151] and the Warwick-Edinburgh Mental Wellbeing Scale [152]. Quality of life indicators, such as the EQ Health and Wellbeing, which include subjective well-being questions, can also be used [153]. Objectively measured (predicted) indicators, such as the OECD Better Life Index, use different dimensions to predict well-being [154].

However, measuring well-being’s multifaceted and dynamic nature is complex, and studies vary widely in methods and scales. The World Database of Happiness summarizes multiple definitions and measurements of well-being [155]. Although there is an increasing trend to measure well-being in surveys, data are neither as readily available nor as consistently measured as mortality data.

### Welfare Economic Analyses

Health and transport economics approaches are widely used within a welfare microeconomic framework. Health economics typically uses cost-effectiveness analysis to assess the cost per health gain, such as Quality-Adjusted Life Years (QALYs). This approach supports interventions that meet specific cost-effectiveness thresholds. In contrast, transport economics employs Cost-Benefit Analysis (CBA), which converts costs and benefits into monetary units. This method balances numerous benefits and costs, including monetised health benefits often obtained through willingness-to-pay surveys. It is acknowledged that CBA outcomes depend on assumptions, the parameters considered, and the unit costs assigned [156].

CBA is mandatory for large transport projects in the EU and UK, assigning monetary values to impacts to determine societal benefits or costs. These analyses often focus on travel time savings despite debates over their validity and equity [157, 158]. This prioritises motorised travel and overactive travel and measures to enable faster motor vehicle journeys. Traditionally, CBA has ignored or downplayed the harms of motor vehicle journeys and inadequately included the benefits of active travel. More generally, challenges persist in using CBA, including insufficient impact data, incomplete parameter consideration, the absence of market values, and the potential incommensurability of items. Critics also highlight CBA’s reliance on neoclassical economic frameworks and assumptions about decision-making, the future, the environment, and fairness [159].

As a decision-theoretic approach, CBA can produce biased results due to omissions (e.g., mental and well-being health is often missing). However, the incommensurability critique of CBA argues that not all values can or should be compared when the starting point is current market prices, income, and wealth. This critique questions the rationale of assigning monetary values to diverse impacts, such as environmental preservation, human health, cultural heritage, and social well-being [160, 161].

CBA is based on the preferences of currently living individuals. It assumes that atomised individuals are ideal decision-makers and that market prices are generally good social value indicators, with the logic of utility maximisation consumption decisions relevant to individuals and societies. Market efficiency presumes individuals are consistent and rational decision-makers with complete information who optimise utility without cognitive biases [162] emotional influences, social conditioning, or limited rationality [163–165].

In practice, markets favour those with more resources, making power imbalances the norm rather than the exception. Determining whose interests are relevant and how to weigh them is crucial; prices based on willingness to pay inherently favour the wealthy.

Future generations and non-human interests are critically important for addressing systemic issues like climate change and environmental sustainability. Evaluations should incorporate intergenerational equity to account for the long-term impacts over millennia, recognising our critical responsibility to preserve environmental limits and ensure future well-being.

An alternative approach would focus on fulfilling objective needs and promoting human flourishing rather than solely emphasising consumption and willingness-to-pay metrics. Approaches such as Multicriteria Decision Analysis (MCDA) capture a broader range of impacts, including qualitative and non-monetizable factors, thereby enhancing the inclusivity of economic assessments.

While policymakers typically are interested in monetary outcomes, fiscal outcomes or local job creation can be of greater policy relevance than welfare economic and monetary equivalents. Macroeconomic assessments of built environment interventions consider broader economic impacts and systemic changes, such as labour market adjustments and technological advancements. They evaluate ripple effects on national or regional economies, including employment, productivity, and growth. However, most macroeconomic models typically adopt a neoclassical economic paradigm, using representative agents and assuming general equilibrium [166], viewing taxes or government interventions as inefficiencies [167] and without consideration of broader social impacts [168].

### Approaches to Uncertainty and Sensitivity Analysis

One challenge relevant to CBA but more broadly to HIA is that these methods typically aim to estimate a single “most plausible” result given current knowledge, often overlooking the low-probability, high-impact risks associated with an unstable climate and potential extinction events. Overcoming this limitation necessitates decision-making frameworks that prioritize resilience and risk mitigation.

Models must capture the range of possible outcomes or conclusions consistent with current knowledge and the likelihood of those outcomes. This is the domain of uncertainty and sensitivity analysis. Uncertain assumptions are often represented as parameters within models, with probability distributions assigned to represent plausible values. This approach has been used to model uncertainty in the impacts of built environment changes on physical activity and air pollution inhalation [169].

By sampling from distributions around multiple input parameters, interval estimates around results can be produced. In many modelling studies, uncertainty analyses are only done where the published data on model inputs includes measures of uncertainty since quantifying other uncertainties can be difficult. For example, confidence intervals are published for exposure-response functions. However, these do not generally account for uncertainties about how the exposure-response function generalises from one population to another or about exposure estimation.

A constructive perspective gauges the potential importance of each source of uncertainty and how the model might be improved with better data. The simplest approach is one-way sensitivity analysis, where results are compared under different plausible assumptions about true parameter values (e.g. what if the PM_2.5_ concentration were 11–15 µg?). If the result is not sensitive to the input, we conclude there is no need to know that input better. However, one-way sensitivity analysis can be cumbersome if there are many uncertain parameters where parameters are correlated or have nonlinear effects. A more formal approach that addresses this limitation is the Value of Information analysis [170]. This aims to quantify the expected benefit of obtaining better information about inputs to a model. Reductions in uncertainty around model outputs can measure benefit. For example, if the expected benefit from perfect information about a parameter is low, then further research about that parameter would not be worthwhile. Value of Information analyses can also determine the expected benefits from performing a study of a particular design and sample size to obtain data about an uncertain quantity. While Value of Information calculations can be performed efficiently with accessible software, they require uncertainties to be parameterised with probability judgements.

### Equity and Justice in HIA

Most health impact modelling studies of transport have not adequately considered equity and distributional aspects [6, 17]. Inequities in health outcomes can be conceptualised as occurring due to differences in (1) exposure, (2) disease burden, (3) susceptibility, and (4) capacity to benefit from an intervention. In addition, it is important to recognise inequities in (5) non-health outcomes (often financial), (6) responsibility for harm, and (7) participation in the HIA process.

In car-dominant societies, benefits primarily accrue to wealthier car-users, while the harms caused by motor vehicles disproportionately affect poorer groups, the young and old, women, and ethnic minorities [12]. Disadvantaged groups often experience environmental and spatial disadvantages resulting in higher exposure to air and noise pollution [171, 172] and road travel injuries [173]. Net Zero interventions have the potential to reduce inequalities [174].

Inequities often result in a higher disease burden (and so greater absolute risk) and greater susceptibility to serious consequences due to the higher prevalence of pre-existing health conditions [175], in part the consequence of other aspects of disadvantage. To benefit from an intervention often requires agency, time, or money. Place-based interventions require the ability to stay in the place, with renters at risk of gentrification. Understanding the household budgetary implications of policies is crucial; methods for environmental HIA could be integrated with those used to study the tax and benefit system [129].

Explicitly including relevant population subgroups and adopting intersectional frameworks throughout assessment stages are necessary to address equity in HIAs. These inequalities occur within a single setting but also between places and intergenerationally, particularly in the context of climate change and environmental limits. Including the needs of future generations requires, firstly, the involvement of those generations already present, but it also requires a different way of thinking in which people collectively consider decisions rather than being aggregated as individual consumers.

Relational power analyses can distinguish between both direct responsibility for environmental harms (e.g., vehicle miles driven) and indirect responsibility through agency in social and economic systems (e.g., pension investments). Pathways are complex, and the disadvantages are multidimensional, requiring specific descriptions of harm distribution and responsibility [176].

HIA frameworks should consider commercial determinants of health, similar to assessments for tobacco and diet, examining how commercial actors influence health and equity, from shaping political and economic systems, controlling data to driving the consumption of harmful products [177].

## Reporting Checklists and Reproducibility

A significant gap in quantitative HIA and simulation studies is the lack of standardised reporting checklists. Such checklists are widely used and supported in primary studies, systematic reviews [178], and related fields like health economics [179]. They are also being introduced for modelling studies on estimating and reporting population health effects resulting from climate change mitigation actions [180]. This lack of standardisation in HIA makes it challenging to conduct bias checking, meta-analyses, and further translation of findings into policy or practice. Without unifying frameworks, comparing results across studies or synthesising evidence becomes difficult, ultimately undermining the credibility and utility of HIA.

To address this gap, part of the standardised reporting framework should focus on reproducibility, which is increasingly recognised as a cornerstone of scientific rigour [181]. For quantitative HIA, this means ensuring the entire analytical process is transparent and accessible. This involves making code openly available on platforms like GitHub (e.g., *ITHIM-R *[13])), accompanied by sample datasets, allowing others to test and validate the analysis and detailed documentation that explains methodologies, assumptions, data sources, and instructions for running the code. Funders should support the time required for this level of documentation, as it is integral to ensuring reproducibility.

Methods for verifying results for consistencies in exposure and outcome changes, sensitivity analyses, and validation against external datasets should also be included to ensure findings are robust and not artefacts of specific assumptions or data inputs. Model developers should compare their models and results to earlier studies to identify differences and understand their drivers. This fosters an iterative approach that strengthens the validity of new models and contributes to a cumulative body of knowledge.

The HIA community can move toward greater transparency, consistency, and reproducibility by adopting these practices. Standardised reporting checklists should explicitly incorporate these elements, ensuring that future studies are methodologically sound and accessible to others. This will enhance the credibility of HIA outputs and facilitate their translation into evidence-based policy and practice.

## Discussion

### State of Research

Quantitative HIA of urban transport and land use has developed rapidly over the last 15 years. The field now draws on a wide range of conceptual frameworks, exposure assessment methods, and modelling approaches. Substantial advances include:

- The use of high-resolution exposure surfaces from satellite data, land use regression, and chemical transport models.
- The integration of behavioural modelling with health outcomes, supported by microsimulation and agent-based methods.
- The development of tools such as ITHIM and HEAT, which bring health impact modelling closer to policy application.
- Epidemiological evidence strengthening dose–response functions for air pollution, physical activity, and noise, with emerging evidence for heat and green/blue space. Exposure–response functions are most advanced for air pollution and physical activity, while still emerging for noise and greenspace.
- Increasing attention to equity and distributional impacts, though integration remains uneven across pathways.

### Promises and Limitations

The diversity of methods and models represents a strength: it allows HIAs to be tailored to different questions, scales, and policy contexts. Promising developments include:

- Person-centric approaches that combine exposure and behaviour at fine spatial and temporal resolution.
- Well-being and economic metrics that extend beyond mortality and morbidity.
- Uncertainty analyses that begin to quantify the robustness of results.

However, limitations remain evident:

- Many HIAs still rely on CRA. While suitable for estimating the burden of a given exposure, CRA cannot represent time dynamics such as changes in disease prevalence, competing causes of death, or indirect effects of morbidity transitions. This creates a tension between providing simple, policy-friendly outputs and addressing the inherent complexity and uncertainty of health modelling.
- Equity considerations are often mentioned but not systematically integrated across pathways or models.
- Scenario definitions are sometimes overly simplistic or based directly on policy documents, without behavioural modelling to test the plausibility of changes in travel demand, activity patterns, or land use.
- The breadth of research risks diluting focus, with some topics (e.g. heat, green/blue space) treated in less depth compared with air pollution and physical activity.
- HIAs are based too much on the present, rather than considering a more uncertain and unstable future.

### Remaining Gaps and Future Needs

Despite these advances, critical gaps remain that must be addressed if HIA is to inform equitable and climate-resilient urban policy:

***Data gaps*** *:*

- Longitudinal and harmonised datasets remain scarce, limiting comparability across settings.
- Although remote sensing and modelling have enabled high-resolution air pollution surfaces, HIAs still typically use annual averages, masking variability.
- More attention is needed to person-centric exposures (both indoor and outdoor), particularly for transport microenvironments, to understand inequalities in exposure.

***Exposure assessment gaps*** *:*

- Multi-pollutant models are rarely applied, even though combined exposures better reflect real-world risks.
- For noise, while estimates have improved, there is still a need for more widespread methods to capture noise on minor roads. Similarly to air pollution, person-centric exposures, incorporating travel, activities, and indoor effects, will likely better capture population variability.
- For heat, while evidence on heat hazard sources is established, data on heat exposure, vulnerability, adaptive measures, and human heat resilience are still emerging. There is a need to better link the spatial scale of climate models to that of microclimate environments.
- Eye-green visibility has helped move towards a person-centric approach for green and blue spaces. Future work should aim to disentangle the pathways to health for integrated assessment with other exposures to avoid double-counting, and when, e.g., greening and heat, the effects may be context-specific.
- For physical activity, we need to better estimate the population distributions of behaviours by demographics and methods for estimating how this would change with interventions. Given the nature of physical activity and the non-linear dose-response relationships, microsimulation and behavioural modelling are more common than other environmental risk factors.

**Epidemiological gaps**, including limited evidence for morbidity outcomes, confounding by socio-economic position, and differential susceptibility. Evidence for air pollution and physical activity is strong. Still, exposure–response functions for noise and green/blue space remain limited, and confounding by socio-economic position is often inadequately addressed. Morbidity outcomes such as mental health and chronic diseases require greater attention.

**Modelling gaps**, notably the need for transparent, reproducible models that integrate behavioural dynamics and allow long-term scenario testing.

- Transparent, reproducible models with open code and documentation are needed to build trust and comparability.
- Scenario design should be explicit, documenting stakeholder involvement, and ideally based on data (e.g. from another setting), evidence (e.g. natural experiments), or behavioural models (e.g. agent-based approaches).
- Few HIAs test robustness under long-term climate scenarios; sensitivity analyses under worsening climatic conditions should become standard to ensure resilience of findings.
- Microsimulation and agent-based models offer opportunities to represent heterogeneous populations, behaviours, and exposures in dynamic environments, but their application to HIA is still in its early stages.

#### Metric Gaps

Outcomes beyond mortality, such as well-being and quality of life, remain under-represented. Methods to incorporate wider costs and benefits (e.g., cost-benefit analysis, welfare economics) exist but remain contentious. Value of Information (VOI) analysis could be used more widely to prioritise new data collection by identifying which uncertainties matter most.

#### Equity Gaps

Equity remains insufficiently embedded in practice. Agent-based models can explicitly simulate how policies affect heterogeneous groups and therefore hold promise for distributional analysis. More broadly, HIAs should systematically integrate distributional impacts, consider household budget effects, intergenerational equity, and recognise power inequalities, ensuring under-represented groups are engaged in participatory processes.

## Research Agenda

Building on the workshop discussions and synthesis presented here, we propose a forward-looking research agenda for quantitative HIA of urban transport and land use in Europe. The agenda is organised around six interlinked domains: data, exposure assessment, epidemiological evidence, modelling approaches, metrics and uncertainty, and equity and justice. Each domain highlights priorities for advancing both research and practice.

### Data

Robust quantitative HIA requires diverse and high-quality data inputs. Priorities include:

- Improved availability and harmonisation of data on travel behaviour, physical activity, air pollution, noise, heat, and green/blue space across European cities.
- Integration of emerging data sources (e.g. wearables, mobile phone mobility data, satellite products) while addressing privacy and representativeness concerns.
- Longitudinal datasets that can capture changes in exposure and health outcomes over time.
- Linkages between environmental, health, and socio-economic data to enable more granular and intersectional analyses.

### Exposure Assessment

Quantitative HIA relies on accurate estimation of population exposures. Key research needs are:

- Advancing multi-pollutant exposure models that reflect combined effects of air pollution mixtures and interactions with noise, heat, and green space.
- Person-centric exposure assessment, integrating time–activity patterns, transport microenvironments, and indoor/outdoor exposures, with particular attention to inequalities.
- High-resolution urban climate modelling to capture microclimates, including heat and cold stress, vulnerability, adaptive measures, and human resilience.
- Dynamic exposure assessment methods (e.g. GPS, remote sensing) that link individual behaviours to environmental conditions.
- Better noise characterisation, including methods that capture exposures from minor roads and transport corridors.

### Epidemiological Evidence

Quantitative HIA requires strong causal evidence linking exposures and outcomes. Priorities include:

- Updating and refining exposure–response functions with new large-scale meta-analyses, particularly for noise, heat, and green/blue space, where evidence remains limited.
- Addressing confounding and effect modification, especially by socio-economic position, in studies linking environmental exposures to health outcomes.
- Expanding research on morbidity outcomes (e.g. mental health, chronic diseases) and not only premature mortality.
- Developing exposure–response functions for emerging exposures, including transport-related particulate matter composition (e.g. tyre and brake wear).
- Testing stratified and context-specific functions, acknowledging heterogeneity of effects across populations and environments.

### Modelling Approaches

New modelling techniques offer opportunities but also raise challenges. Priorities are:

- Developing typologies of HIA approaches, clarifying the role of different methods (comparative risk assessment, life tables, microsimulation, agent-based modelling).
- Integration of behavioural and exposure models to better capture the determinants of transport behaviour and their implications for health.
- Improving transparency and reproducibility of models, including open-source code, documentation, and validation against external datasets.
- Scenario design standards, ensuring scenarios are explicit, document stakeholder involvement, and are based on data (e.g. natural experiments), evidence, or behavioural models.
- Testing long-term scenarios under conditions of climate change, adaptation, and resilience, with sensitivity analyses for severely worsening conditions.
- Exploring agent-based models as a tool to simulate heterogeneous populations, individual behaviours, and equity outcomes in dynamic environments.

### Metrics and Uncertainty

The interpretation and policy use of HIA depend on the chosen metrics and how uncertainty is handled. Priorities are:

- Broadening outcome metrics to include well-being, quality of life, and economic impacts in addition to morbidity and mortality.
- Systematic treatment of uncertainty, using Monte Carlo simulation and sensitivity analysis to provide credible intervals around estimates.
- Applying Value of Information approaches to identify which uncertainties matter most and where new data collection would be most efficient.
- Applying Value of Information (VOI) approaches, inverting the epidemiology-HIA relationship to identify which uncertainties matter most and where new data collection would be most efficient.
- Developing reporting checklists to standardise methods and improve comparability across studies.
- Assessing trade-offs between simplicity and comprehensiveness, balancing the need for policy-friendly metrics with the complexity of real-world systems.

### Equity and Justice

Equity emerged as a central but insufficiently addressed theme across all domains. Priorities include:

- Systematically integrating equity considerations at all stages of HIA (exposures, susceptibility, disease burden, capacity to benefit, participation).
- Developing intersectional approaches that capture how multiple dimensions of disadvantage interact (e.g. age, gender, ethnicity, socio-economic position).
- Assessing distributional impacts of policies, including household budget implications, risks of gentrification, and intergenerational equity.
- Recognising power inequalities and proactively engaging underrepresented groups in participatory processes.
- Ensuring HIA methods are democratic and transparent, embedding participation in line with broader goals of social justice and sustainability.
- Addressing commercial and political determinants of health, including how transport and planning decisions are shaped by industry actors and power imbalances.

This research agenda reflects consensus among workshop participants and subsequent synthesis by the authors. It emphasises the need for methodological innovation, systematic integration of equity, and long-term perspectives in light of climate change. By organising priorities into six domains, we provide a roadmap for future research that can advance the scientific rigour, policy relevance, and societal impact of quantitative HIA in Europe.

## Data Availability

No datasets were generated or analysed during the current study.
